# Corticosteroid use and increased CXCR2 levels on leukocytes are associated with lumacaftor/ivacaftor discontinuation in cystic fibrosis patients homozygous for the *F508del CFTR* mutation

**DOI:** 10.1371/journal.pone.0209026

**Published:** 2018-12-12

**Authors:** Kerstin Pohl, David P. Nichols, Jennifer L. Taylor-Cousar, Milene T. Saavedra, Matthew J. Strand, Jerry A. Nick, Preston E. Bratcher

**Affiliations:** 1 Department of Medicine, National Jewish Health, Denver, CO, United States of America; 2 Department of Pediatrics, University of Washington, Seattle, WA, United States of America; 3 Department of Pediatrics, National Jewish Health, Denver, CO, United States of America; 4 University of Colorado School of Medicine, Aurora, CO, United States of America; 5 Biostatistics and Bioinformatics, National Jewish Health, Denver, CO, United States of America; University of Alabama at Birmingham, UNITED STATES

## Abstract

Cystic fibrosis (CF) is the most common life-shortening genetic disease and is caused by mutations in the cystic fibrosis transmembrane conductance regulator (CFTR) gene. Several current therapies aim at improving availability and/or function of the mutant CFTR proteins. The combination therapeutic lumacaftor/ivacaftor (Orkambi, luma/iva) partially corrects folding and potentiates CFTR function impaired by the *F508del* mutation. Despite the potential for clinical benefit, a substantial number of patients discontinue treatment due to intolerable adverse effects. The aim of the present study is to identify differences between individuals who continued treatment and those who discontinued due to adverse respiratory effects to potentially inform treatment decisions. Clinical data from the year prior to treatment initiation were analyzed from 82 patients homozygous for the *F508del* mutation treated at the Colorado Adult CF Program. Blood samples were collected from 30 of these subjects before initiation of treatment to examine expression of circulating leukocyte surface antigens and cytokines. Clinical and demographic characteristics were analyzed along with inflammatory markers to determine biomarkers of drug discontinuation. The use of oral prednisone and/or nasal budesonide in the year prior to luma/iva initiation was more prevalent in CF subjects who did not tolerate luma/iva (82% vs. 43%). Increased age, but not gender or initial lung function, was associated with higher probability of discontinuing treatment due to side effects overall. Worse lung function (lower ppFEV_1_, ppFEF_25-75_ ≤ 60%) was associated with higher incidence of discontinuing treatment due to pulmonary adverse effects. In a nested cohort of patients, increased surface levels of CXCR2 on CD14+CD16- monocytes were associated with discontinuation. Overall, the patients who tolerated luma/iva were distinguishable from those who did not tolerate the drug based on clinical and cellular markers obtained prior to treatment initiation.

## Introduction

Cystic fibrosis (CF) is a disease resulting from defective ion transport caused by mutations in the cystic fibrosis transmembrane conductance regulator (CFTR) gene [1]. CFTR is a membrane chloride channel [2], which also transports bicarbonate [3] and glutathione anions [4]. Defective CFTR function in airway epithelial cells is the primary cause for morbidity and mortality in CF. In addition to the airways, CFTR is expressed in the epithelial cells of many organs [5], and CFTR dysfunction can have significant impacts on these systems, most notably the gastrointestinal tract and pancreas.

A current emphasis of CF therapeutic development is the use of small molecules to target CFTR protein dysfunction directly. Collectively referred to as CFTR modulators, these compounds are designed to increase cell surface levels and/or correct function of the mutated CFTR protein. CFTR correctors, including lumacaftor (luma, VX-809), aid in the processing of F508del mutant CFTR protein and increase its cell membrane expression [6]. CFTR potentiators, such as ivacaftor (iva, VX-770, Kalydeco), increase the open probability of CFTR proteins with dysfunctional channel gating [7–9]. The combination of lumacaftor/ivacaftor (luma/iva, Orkambi) was approved in the U.S. in 2015 for treatment of patients homozygous for the *F508del* mutation [10]. In vitro, this drug combination was shown to improve F508del CFTR folding and function to 35% of normal CFTR channel activity [6]. In phase 3 trials, patients receiving luma/iva therapy averaged a 2.8% increase in forced expiratory volume in first second in percent predicted (ppFEV_1_) compared to those receiving placebo, and the frequency of exacerbations was reduced by 39% in the treated group [11]. An open-label follow-up study (NCT01931839) has recently been completed and preliminary results support sustained lower annual rates of exacerbations and slower decline in ppFEV_1_ over the same time period compared to untreated matched controls in the US CF Patient National Registry [12].

The inability of a subpopulation of *F508del* homozygous patients to tolerate luma/iva treatment is of significant clinical interests. While chest tightness was a common complaint during the clinical trials and clinical use, most patients report resolution of these symptoms within 2 weeks and only 5% discontinued treatment [13]. However, clinical experience worldwide has confirmed a significantly larger percentage of patients whose symptoms resulted in discontinuation of the medication [14, 15]. In a recent study, a large U.S. CF program reported 26.7% of patients prescribed luma/iva failed to tolerate full dose therapy [14].

Given the high frequency of adverse respiratory effects and drug discontinuation, we retrospectively analyzed data sets collected from subjects before initiation of luma/iva treatment in order to identify clinical parameters as well as markers of inflammation associated with higher rates of drug intolerance.

## Materials and methods

### Study design and patient recruitment

**Fig 1:**
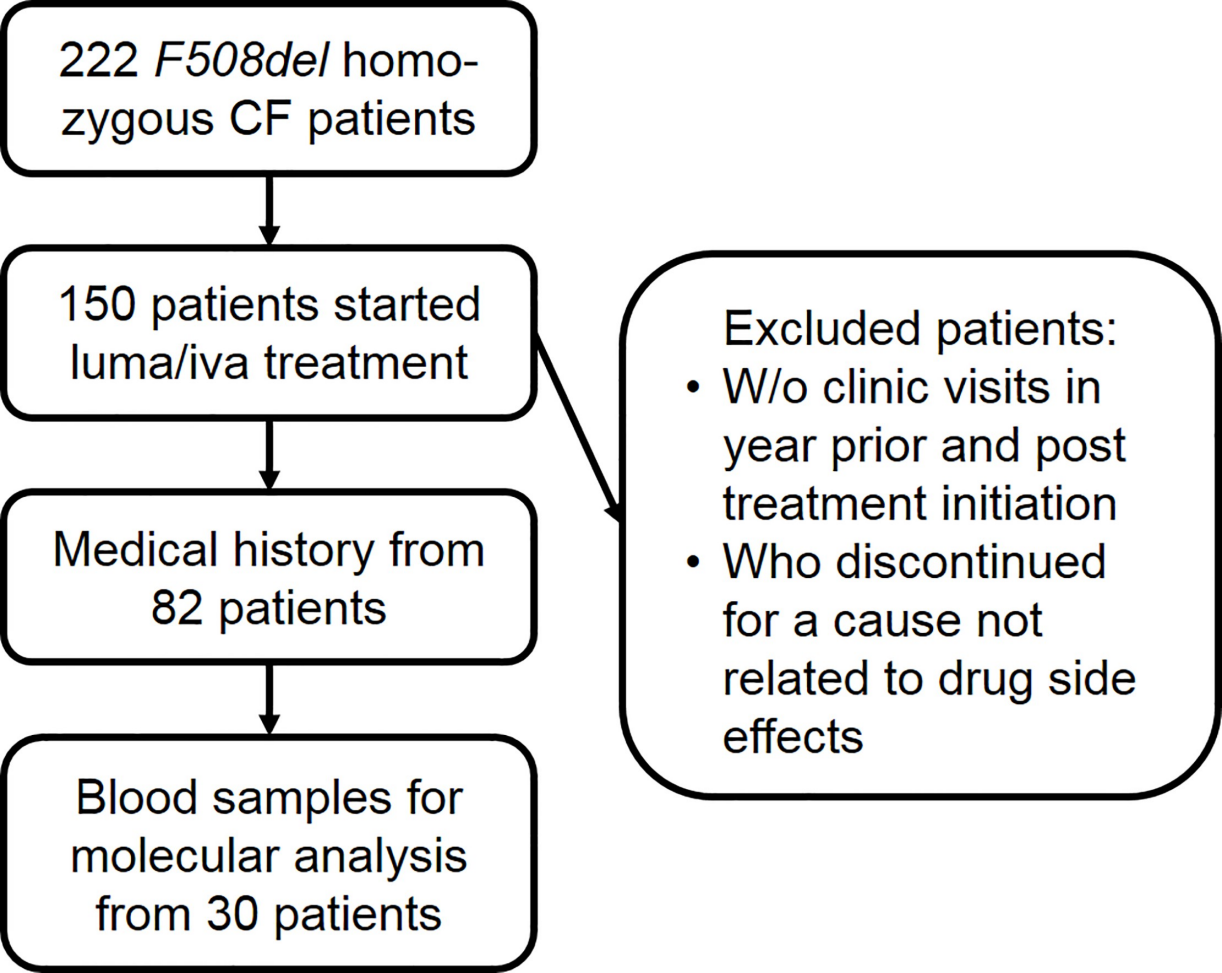
Schematic overview of patient recruitment from the Colorado Adult CF program.

Clinically stable CF patients with genotype *F508del/F508del* who have been prescribed twice-daily lumacaftor 400 mg/ivacaftor 250 mg therapy (Orkambi) were recruited between July 2015 and June 2016. Of 222 CF patients with this genotype in the Colorado Adult CF Program, 150 patients started luma/iva treatment (Fig 1). Patients whose complete medical record was not available for the year prior and post initiation, or who discontinued for a cause not related to medication side effects (insurance, pregnancy, and antifungal or antimycobacterial treatment) were excluded from this study. In total, medical information was analyzed from 82 patients. In addition, blood samples were collected from 30 of these subjects before initiation of treatment. Healthy control volunteers (n = 5) were without respiratory disease. Ethical approval for collection of blood samples and medical information from healthy volunteers and CF patients was obtained from National Jewish Health Institutional Review Board, and all blood donors gave written informed consent.

### Blood sample collection and plasma analysis

Peripheral blood samples were collected by venipuncture into heparinized tubes and immediately spun down at 350x g for 20 min to separate cells from plasma. Remaining debris was removed by spinning plasma samples at 1000x g for 10 min. Chemokines and neutrophil proteins in plasma samples were quantified by various immunoassays. Enzyme-linked immunosorbent assays (ELISAs) were carried out according to manufacturer’s protocol to measure neutrophil elastase, soluble TNF receptor 1 (sTNFR1, both abcam), and IL-8 (R&D Systems).

### Flow cytometry

For baseline quantification of surface receptors, blood leukocytes were stained and analyzed using flow cytometry as previously described [16]. Whole blood samples were collected into K_2_EDTA vacutainers (BD Biosciences) during the same blood draw described above. Samples were centrifuged at 400x g for 10 min at 4°C, plasma was removed, and cells were resuspended to the original volume in ice cold PBS containing 2.5 mM EDTA. Cells were kept on ice and in the dark for the remaining steps. Cells were incubated with a live/dead discriminator (Life Technologies) and an Fc-blocking reagent (TruStain FcX, BioLegend) for 15 min before an antibody cocktail was added for an additional 15 min. The antibody cocktail consisted of antibodies against CD16 (clone 3G8, BioLegend), CD14 (clone 61D3, eBioscience), CXCR2 (clone 5E8-C7-F10, eBioscience), activated CD11b (clone CBRM1/5, eBioscience), CD63 (clone H5C6, eBioscience), and CD66b (clone G10F5, eBioscience). Live, singlet cells were analyzed using a LSR Fortessa flow cytometer (BD Biosciences) and gated based on forward scatter, side scatter, and surface marker expression (neutrophils are defined as CD16^High^ FSC^High^ SSC^Mid^, monocytes as CD14^High^ CD63^High^ FSC^Mid^ SSC^Mid^, and NK cells as CD16^Mid^ FSC^Low^ SSC^Low^).

### Statistical analysis

Results are presented as mean ± standard error of the mean (SEM) of n separate biological replicates as stated in the figure legends. GraphPad Prism (version 6.07 for Windows) was employed for statistical analysis including the frequency distribution analyses (with contingency tables) employing Fisher’s exact test. To determine whether data was normally distributed the D'Agostino and Pearson omnibus normality test was carried out and Grubb’s test was used to detect outliers. When normally distributed or for comparison of small datasets without outliers (n < 6) [17], groups were compared by Student’s t test, otherwise by the nonparametric Mann-Whitney U test. P values were considered statistically significant if P < 0.05. Logistic regression analyses were performed using XLSTAT software (version 2017.1 in Excel 2013 for Windows 7).

## Results

### Prior steroid use is more frequent in individuals who discontinued use of luma/iva

To investigate potential differences in clinical parameters between individuals who continued luma/iva and those who discontinued use, *F508del* homozygous patients with complete medical histories available and who started luma/iva treatment at the Colorado Adult CF Program (n = 82) were included (Fig 1). In total, 17 individuals (20.7%) discontinued use due to intolerable adverse effects with the main complaints being chest tightness (n = 11), which was sometimes associated with a significant drop in ppFEV_1_ (3/11), and gastrointestinal symptoms including diarrhea (n = 2), abdominal pain (n = 1) and nausea (n = 1). Other rare adverse effects (n = 1 each) included pericarditis, dysphagia, allergy, possible Stevens-Johnson syndrome and high liver function test results.

Routinely measured CF-relevant clinical parameters available for all 82 patients were analyzed. Patient demographics, including lung function and BMI at the time of treatment initiation and sex were similar for subjects who stopped or continued treatment (Table 1). Furthermore, incidence of CF-related diabetes and pathogens detected in the year prior to luma/iva initiation were comparable. Individuals who continued treatment were significantly younger (by 5.5 years, p = 0.042) and used less nasal corticosteroids (budesonide and/or fluticasone; 51% relative difference, p = 0.044) in the prior year.

**Table 1:** Baseline patient characteristics, and diagnostic and treatment history for the year prior to luma/iva initiation.

	*F508del* homozygous patients (n = 82)		
	Continued luma/iva	Stopped luma/iva	P value
Number of patients, n (%)	65 (79%)	17 (21%)	
Males, n (%)	37 (57%)	10 (59%)	1.000
**Age at treatment initiation (years)**	**30.2 (28.4–31.9)**	**35.7 (30.0–41.3)**	**0.042**
Lung function values:			
- ppFEV_1_ (%)	67.1 (61.0–73.3)	64.9 (50.7–79.2)	0.754
- ppFEV_1_ ≤ 40%	12 (19%)	4 (24%)	0.733
- ppFEV_1_ below baseline?	17 (26%)	3 (18%)	0.545
- ppFEF_25-75_ (%)	43.7 (36.8–50.6)	45.7 (27.1–64.2)	0.827
- ppFEF_25-75_ ≤ 60%	46 (71%)	12 (71%)	1.000
BMI (kg/m^2^)	21.7 (21.0–22.3)	22.2 (20.9–22.3)	0.508
PMN (mio/ml of blood)	7.9 (7.2–8.6)	7.3 (6.0–8.6)	0.384
CFRD positive, n (%)	29 (45%)	9 (53%)	0.593
At least one positive sputum culture, n (%):			
- non-mucoid PA	48 (74%)	13 (76%)	1.000
- mucoid PA	34 (52%)	9 (53%)	1.000
- NTM MAC	9 (14%)	2 (10%)	1.000
- NTM MABC	6 (9%)	1 (5%)	1.000
Treatment in prior year, n (%):			
- Systemic prednisone	25 (39%)	9 (53%)	0.407
- **Nasal corticosteroids**	**17 (26%)**	**9 (53%)**	**0.044**
- Inhaled corticosteroids	34 (52%)	5 (29%)	0.109
- Inhaled β2 receptor agonists	52 (80%)	12 (71%)	0.511
- Sinus surgery	5 (8%)	2 (12%)	0.631
- Exacerbation, IV Ab	41 (63%)	13 (76%)	0.395

Values are presented as mean (95% confidence interval of mean) or numbers and percentages where indicated. P values were calculated by two-tailed t test and Fisher’s exact test for continuous and categorical variables, respectively. Significant parameters are highlighted in bold.BMI, body mass index; CFRD, CF-related diabetes; ppFEF25-75, forced expiratory flow at 25–75% of the pulmonary volume in percent predicted; ppFEV1, forced expiratory volume in first second in percent predicted; IV Ab, patient with at least one exacerbation requiring intravenous antibiotics in the prior year; MAC, Mycobacterium avium complex; MABC, Mycobacterium abscessus complex; NTM, non-tuberculous mycobacteria; PA, Pseudomonas aeruginosa; PMN, circulating neutrophils.

When patients who discontinued treatment were further stratified by pulmonary versus other adverse events, further differences in clinical parameters became apparent (Table 2). Patients who discontinued due to pulmonary adverse effects were on average 8.2 years older than patients who continued (38.4 vs. 30.2, P = 0.007). In addition, they exhibited slightly lower lung function (ppFEV_1_ 56.8% vs. 67.1%, ppFEF_25-75_ 31.6% vs. 43.7%), higher rate of exacerbations requiring hospitalization (91% vs. 63%) and higher use of systemic and nasal, but not inhaled corticosteroids, compared to people who continued treatment. Compared to patients who discontinued due to other adverse effects, patients with pulmonary side effects had lower lung function (ppFEV_1_ 56.8% vs. 79.8%, ppFEF_25-75_ 31.6% vs. 71.3%), with higher proportion of patients with ppFEV_1_ and ppFEF_25-75_ below 40% and 60%, respectively (27% vs. 17% and 91% vs. 33%).

**Table 2:** Patient clinical history for the year prior to luma/iva initiation stratified by adverse effects.

	*F508del* homozygous patients (n = 82)				
	Continued luma/iva	Discontinued luma/iva (n = 17, 21%)		P values	
		Pulmonary adverse effects	Other adverse effects	Continued vs. Pulmonary AE	Pulmonary vs. other AE
Number of patients, n (%)	65 (79%)	11/17 (65%)	6/17 (35%)		
Age at treatment initiation (years)	**30.2 (28.4–31.9)**	**38.4 (31.4–45.4)**	30.7 (19.1–42.3)	**0.007**	**0.171**
Lung function values:					
- ppFEV_1_ (%)	67.1 (61.0–73.3)	56.8 (41.0–72.7)	79.8 (47.6–112.0)	0.201	**0.104**
- ppFEV_1_ ≤ 40%	12 (19%)	3/11 (27%)	1/6 (17%)	**0.446**	1.000
- ppFEV_1_ below baseline?	17 (26%)	2/11 (18%)	1/6 (17%)	**0.720**	1.000
- ppFEF_25-75_ (%)	43.7 (36.8–50.6)	31.6 (17.7–45.6)	71.3 (23.4–119.3)	**0.173**	**0.062**
- ppFEF_25-75_ ≤ 60%	46 (71%)	**10/11 (91%)**	**2/6 (33%)**	**0.270**	**0.028**
Treatment in prior year, n (%):					
- Systemic prednisone	25 (39%)	7/11 (63%)	2/6 (33%)	**0.186**	**0.335**
- Nasal corticosteroids	17 (26%)	6/11 (55%)	3/6 (50%)	**0.079**	1.000
- Inhaled corticosteroids	34 (52%)	4/11 (36%)	1/6 (17%)	**0.516**	**0.600**
- Inhaled β2 receptor agonists	52 (80%)	9/11 (82%)	3/6 (50%)	1.000	**0.280**
- Exacerbation, IV Ab	41 (63%)	10/11 (91%)	3/6 (50%)	**0.090**	**0.099**

Values are presented as mean (95% confidence interval of mean) or numbers and percentages where indicated. P values were calculated by two-tailed t test and Fisher’s exact test for continuous and categorical variables, respectively. Significant parameters are highlighted in bold and underlined. Non-significant parameters that differed by more than 30% from patients who discontinued due to pulmonary AE, are highlighted in bold. AE, adverse effects; ppFEF25-75, forced expiratory flow at 25–75% of the pulmonary volume in percent predicted; ppFEV1, forced expiratory volume in first second in percent predicted; IV Ab, patient with at least one exacerbation requiring intravenous antibiotics in the prior year.

### Development of a model using varying parameters to distinguish between subjects who continued luma/iva and those who discontinued use due to pulmonary adverse effects

**Fig 2:**
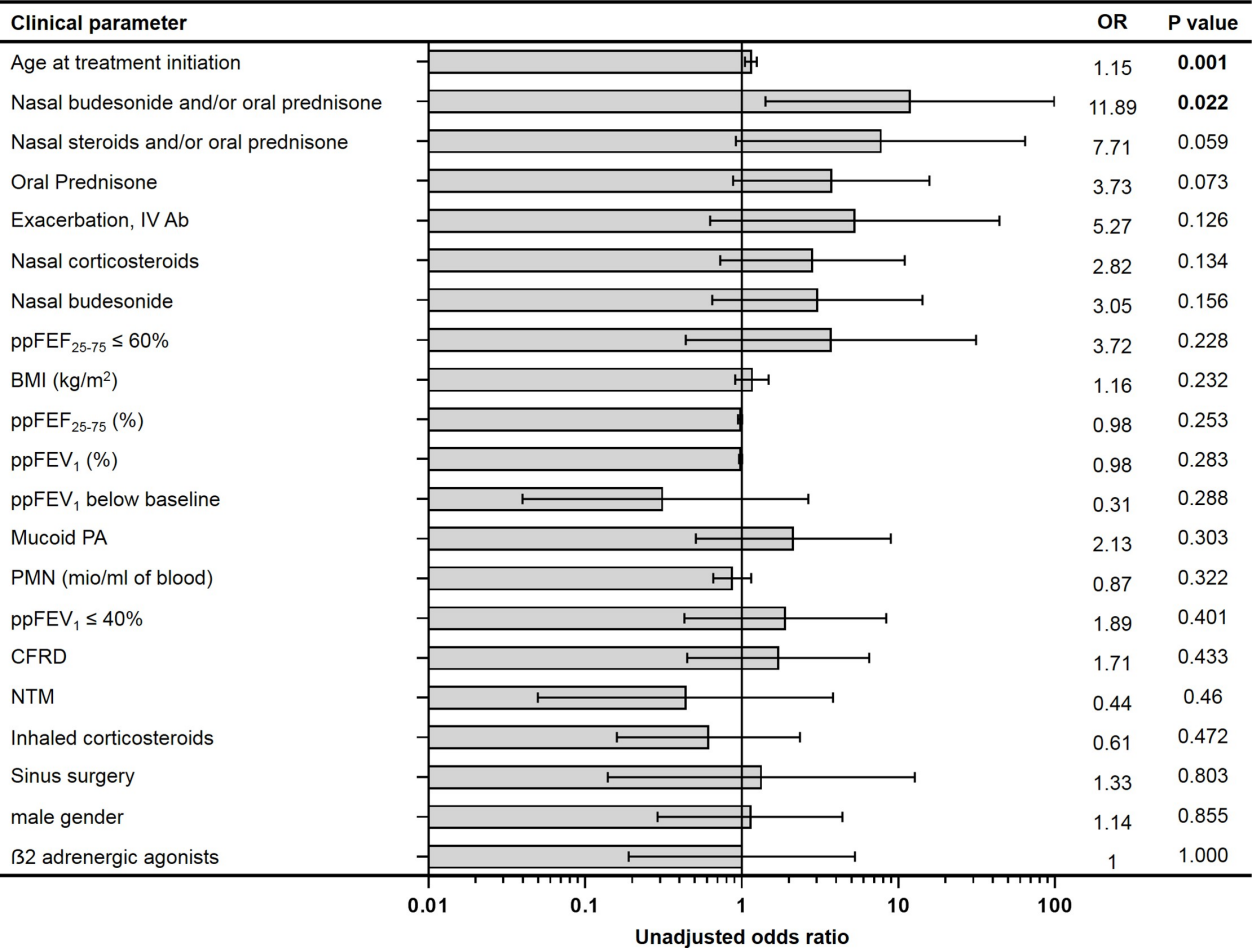
Unadjusted odds ratios (OR) of all recorded clinical parameters associated with treatment discontinuation due to pulmonary adverse effects. Unadjusted odds ratios (OR), lower (-95%) and upper bounds (+95%) of 95% confidence interval and P values are shown. Variables are sorted from higher to lower influence for distinguishing individuals who discontinued treatment due to pulmonary adverse effects as indicated by smaller to greater P values. Significant parameters are highlighted in bold. BMI, body mass index; CFRD, CF-related diabetes; ppFEF_25-75_, forced expiratory flow at 25–75% of the pulmonary volume in percent predicted; ppFEV_1_, forced expiratory volume in first second in percent predicted; IV Ab, exacerbation requiring intravenous antibiotics; NTM, non-tuberculous mycobacteria; OR, odds ratio; PA, *Pseudomonas aeruginosa*; PMN, circulating neutrophils; Pred/bud, oral prednisone and/or nasal budesonide.

Based on the differences observed in clinical parameters between subjects who continued or stopped luma/iva treatment due to pulmonary adverse effects, logistic regression analysis was carried out to develop a model for identifying patients at increased risk for treatment discontinuation. Of all evaluated parameters, only the unadjusted odds ratios of age at treatment initiation and use of oral prednisone and/or nasal budesonide were statistically significant at 1.15 and 11.89, respectively (P = 0.001 and P = 0.022, Fig 2).

To develop a model with the highest accuracy in distinguishing individuals who discontinued drug treatment due to pulmonary side effects, backwards selection of parameters was performed by stepwise removal of the least significant parameter (highest P value) (Table 3). The accuracy of the resulting model is 93.3% with specificity (true designation of continuation) and sensitivity (true designation of discontinuation due to pulmonary adverse effects) of 96.9% and 70.0%, respectively.

**Table 3:** Logistic regression model for distinguishing individuals who discontinued treatment due to pulmonary adverse effects employing clinical parameters.

Model name	All	All-1	All-2	All-3	All-4	All-5	All-6	All-7	All-8	All-9
**AIC**	58.1	56.1	54.2	52.3	51.2	50.1	**48.7**	47.6	46.7	45.7
**-2 log (likelihood) P**	0.0070	0.0042	0.0025	0.0014	0.0011	0.0008	**0.0005**	0.0003	0.0002	0.0001
**Hosmer-Lemeshow P**	0.127	0.117	0.122	0.121	0.099	0.804	**0.429**	0.990	0.982	0.878
**Specificity**	96.90%	96.90%	96.90%	96.90%	96.90%	96.90%	**96.90%**	95.40%	93.80%	93.80%
**Sensitivity**	60.00%	70.00%	60.00%	60.00%	60.00%	50.00%	**70.00%**	50.00%	40.00%	40.00%
**Accuracy**	92.00%	93.30%	92.00%	92.00%	92.00%	90.70%	**93.30%**	89.30%	86.70%	86.70%
**Clinical parameters:**	Age	Age	Age	Age	Age	Age	**Age**	Age	Age	Age
	Pred/bud	Pred/bud	Pred/bud	Pred/bud	Pred/bud	Pred/bud	**Pred/bud**	Pred/bud	Pred/bud	Pred/bud
	ppFEF_25-75_ ≤60%	ppFEF_25-75_ ≤60%	ppFEF_25-75_ ≤60%	ppFEF_25-75_ ≤60%	ppFEF_25-75_ ≤60%	ppFEF_25-75_ ≤60%	**ppFEF**_**25-75**_ **≤60%**	ppFEF_25-75_ ≤60%	ppFEF_25-75_ ≤60%	ppFEF_25-75_ ≤60%
	ppFEV_1_	ppFEV_1_	ppFEV_1_	ppFEV_1_	ppFEV_1_	ppFEV_1_	**ppFEV**_**1**_	ppFEV_1_	ppFEV_1_	ppFEV_1_
	ppFEV_1_ below baseline	ppFEV_1_ below baseline	ppFEV_1_ below baseline	ppFEV_1_ below baseline	ppFEV_1_ below baseline	ppFEV_1_ below baseline	**ppFEV**_**1**_ **below baseline**	ppFEV_1_ below baseline	ppFEV_1_ below baseline	
	CFRD	CFRD	CFRD	CFRD	CFRD	CFRD	**CFRD**	CFRD		
	PMN	PMN	PMN	PMN	PMN	PMN	**PMN**			
	BMI	BMI	BMI	BMI	BMI	BMI				
	Exac	Exac	Exac	Exac	Exac					
	ppFEV_1_ ≤40%	ppFEV_1_ ≤40%	ppFEV_1_ ≤40%	ppFEV_1_ ≤40%						
	NTM	NTM	NTM							
	PA M	PA M								
	ppFEF_25-75_									

The most accurate identification model based on the clinical parameters of 76 patient was generated by backward selection. The least significant parameters were removed one by one from the initial model that included the most significant clinical parameters (model “All”) until AIC (Akaike information criterion) reached a minimum (models ranked by AIC). The AIC and P values of the likelihood ratio test and the Hosmer-Lemeshow test are estimators of the relative quality (goodness of fit) of statistical models; the lower these values, the better the model compared to the other models. The most important criterion of a model is the accuracy in specificity (true identification of continuation) and sensitivity (true identification of discontinuation due to pulmonary adverse effect). The highest, second highest and third highest percentages for each statistical measure are highlighted in dark, medium and light gray, respectively. Based on overall accuracy and significance, model “All-6” (highlighted in bold) was selected for further evaluation. AIC, Akaike information criterion; BMI, body mass index; CFRD, CF-related diabetes; Exac, exacerbation requiring intravenous antibiotics; ppFEF25-75, forced expiratory flow at 25–75% of the pulmonary volume in percent predicted; ppFEV1, forced expiratory volume in first second in percent predicted; NTM, non-tuberculous mycobacteria; PA M, mucoid Pseudomonas aeruginosa; PA NM, non-mucoid Pseudomonas aeruginosa; PMN, circulating neutrophils; Pred/bud, oral prednisone and/or nasal budesonide.

The adjusted odds ratios (OR) and P values for the included parameters are shown in Table 4. The odds ratio of age at treatment start was increased from unadjusted 1.15 to adjusted 1.26, highlighting its association with treatment discontinuation when corrected for other parameters. The frequency of discontinuation was 3.8-fold higher for individuals over 30 years of age (P = 0.10) and 7.7-fold higher for individuals over 35 years of age (P = 0.004) in our patient cohort.

**Table 4:** Adjusted odds ratios (OR) of clinical parameters included for most accurate designation of subjects discontinuing treatment due to pulmonary adverse effects.

Clinical parameter	OR	OR (-95%)	OR (+95%)	P value
ppFEF25-75 ≤ 60%	29.08	0.53	1596.29	0.099
Pred/bud use in prior year	8.61	0.51	61.56	0.159
CFRD	2.67	0.39	18.31	0.317
**Age at treatment initiation**	**1.26**	**1.07**	**1.48**	**0.006**
ppFEV_1_	1.05	0.96	1.11	0.148
PMN at treatment initiation	0.82	0.54	1.24	0.346
ppFEV_1_ below baseline	0.13	0.003	6.42	0.307

Despite the remaining variables not reaching statistical significance, the overall associations indicate that a patient’s risk of discontinuing luma/iva treatment due to pulmonary side effects further increases with having CF-related diabetes (CFRD), oral prednisone and/or nasal budesonide use (pred/bud) in the year prior to treatment start, and a ppFEF_25-75_ below 60%. Conversely, increased neutrophil count and a ppFEV_1_ below the usual baseline might lower the risk of discontinuation.

Despite the remaining variables not reaching statistical significance, the overall associations indicate that a patient’s risk of discontinuing luma/iva treatment due to pulmonary side effects further increases with having CF-related diabetes (CFRD), oral prednisone and/or nasal budesonide use (pred/bud) in the year prior to treatment start, and a ppFEF_25-75_ below 60%. Conversely, increased neutrophil count and a ppFEV_1_ below the usual baseline might lower the risk of discontinuation.

### Surface expression of CXCR2 is selectively elevated on peripheral blood leukocytes of subjects who discontinued use of luma/iva

Of the 82 patients whose clinical parameters were analyzed above, peripheral blood was obtained from a subset (n = 30) of these individuals (Fig 1). Demographic and baseline characteristics of this cohort were similar between patients who stopped and continued luma/iva treatment (Table 5), and all individuals were clinically stable at the time of blood collection. The variables with the greatest differences between the groups included age at time of treatment initiation (P = 0.106) and use of systemic (prednisone) and/or inhaled (budesonide) steroids (pred/bud, P = 0.210).

**Table 5:** Baseline patient characteristics, diagnostic and treatment history for the year prior to luma/iva initiation.

	*F508del* homozygous patients (n = 30)		
	Continued luma/iva	Stopped luma/iva	P value
Number of patients, n (%)	22 (64%)	8 (36%)	
Males, n (%)	12 (55%)	6 (75%)	0.419
Age at treatment initiation (years)	28.4 (25.3–31.4)	34.4 (23.8–45)	0.106
ppFEV_1_ (%)	74.7 (64.1–85.3)	67.1 (41.1–93.1)	0.486
BMI (kg/m^2^)	22.4 (21.3–23.6)	23.4 (21.6–25.3)	0.327
PMN (mio/ml of blood)	7.2 (6.1–8.3)	7.5 (4.9–10.1)	0.790
CFRD positive, n (%)	10 (45%)	3 (38%)	1.000
At least one positive sputum culture, n (%):			
- non-mucoid PA	15 (68%)	5 (63%)	1.000
- mucoid PA	11 (50%)	4 (50%)	1.000
- NTM	2 (9%)	0 (0%)	1.000
Treatment in prior year, n (%):			
- Pred/bud	7 (32%)	5 (63%)	0.210
- sinus surgery	1 (5%)	0 (0%)	1.000
- Exacerbation, IV Ab	10 (46%)	4 (50%)	0.887

Values are presented as mean (95% confidence interval of mean) or numbers and percentages where indicated. P values were calculated by two-tailed t test and Fisher’s exact test for continuous and categorical variables, respectively. BMI, body mass index; CFRD, CF-related diabetes; ppFEV1, forced expiratory volume in first second in percent predicted; IV Ab, patient with at least one exacerbation requiring intravenous antibiotics in the prior year; NTM, non-tuberculous mycobacteria; PA, Pseudomonas aeruginosa; PMN, circulating neutrophils; Pred/bud, oral prednisone and/or nasal budesonide.

**Fig 3:**
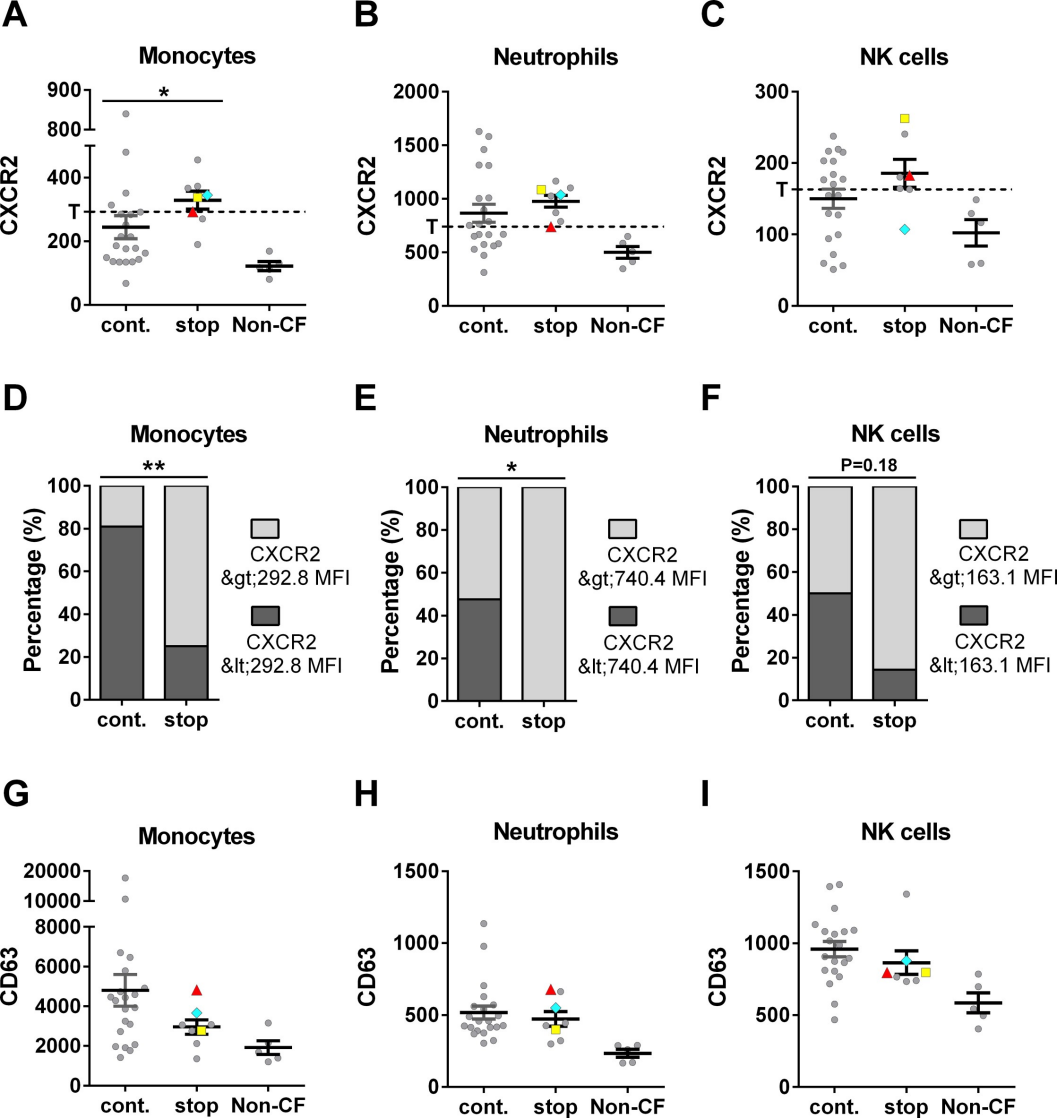
CXCR2 and CD63 expression on circulating leukocytes. Flow cytometry was employed to measure the cell surface expression of CXCR2 (AF) and CD63 (GI) on circulating CD14+ CD16- monocytes (A, D, G), neutrophils (B, E, H), and NK cells (C, F, I) from non-CF and CF subjects who continued (cont.) or discontinued (stop) luma/iva use. All values are median fluorescence intensity (MFI). For the values from subjects who discontinued use, gray circles denote values from subjects who discontinued due to pulmonary adverse effects, a red triangle denotes values from a subject who developed pericarditis, a cyan diamond denotes values from a subject who experienced severe diarrhea, and a yellow square denotes values from a subject who experienced severe abdominal pain. CXCR2 levels on monocytes (A) were significantly different between the CF groups (P = 0.012 by two-tailed Mann-Whitney U test). Frequency distribution analyses (via contingency tables) was performed on CXCR2 data to determine optimal threshold levels (T, dotted lines) for distinguishing individuals who continued drug treatment from those who discontinued. Utilizing CXCR2 as a dichotomous variable (above or below threshold) improved the significance of identifying treatment discontinuation (DF) as CF subjects who discontinued were predominantly above the threshold levels (two-tailed P values by Fisher’s exact test). n≥7 for both CF groups and n = 5 for non-CF controls.

Using a panel of antibodies against markers found to be of interest in a similar study [16], surface marker expression on peripheral blood leukocytes obtained from *F508del* homozygous CF subjects immediately prior to luma/iva treatment and non-CF controls (n = 5) was measured by flow cytometry (Fig 3). CXCR2 is a receptor for IL-8, which is a well-described inflammatory cytokine with roles in CF disease [18], and surface CD63 levels serve as a marker of degranulation indicative of cellular activation [19]. CXCR2 levels on the surface of CD14+ CD16- monocytes, neutrophils, and NK cells were increased in subjects who subsequently did not tolerate luma/iva treatment (n = 8) as compared to subjects who did tolerate the drug (n = 22), but only levels measured on monocytes reached statistical significance (P = 0.012). CXCR2 levels for all CF patients combined were significantly greater compared to non-CF controls (P<0.05). We applied threshold levels to examine CXCR2 levels as a dichotomous variable (above or below threshold) to further distinguish between the two patient groups. Thresholds were set to be greater than healthy control values and to reach the most significant differentiation between drug continuation and discontinuation by Fisher’s exact test. CXCR2 in cells from CF subjects who tolerated luma/iva were more frequently below these threshold levels (Fig 3D–3F). This effect was specific for CXCR2, as surface levels of CD63 were not significantly different between the groups. Interestingly, the cause for treatment discontinuation, pulmonary or other adverse effects, did not affect CXCR2 or CD63 levels (Fig 3). Therefore, patients who discontinued luma/iva were not stratified based on the underlying cause of discontinuation in this cohort.

In addition to these cell surface receptors, plasma concentrations of common inflammatory markers were measured by ELISA (S1 Fig). These markers included IL-8 (a cytokine of known importance in CF [18]), sTNFR1 (a proteolytically shed receptor of TNFα that is increased during systemic inflammation and bacterial pneumonia [20]), and neutrophil elastase (NE) (a well-described marker of inflammation in CF [21]). No significant differences were detected between patients who continued and stopped luma/iva treatment; however, levels of IL-8 and NE in both subgroups analyzed separately or combined (all CF patients) were significantly greater than measured in non-CF controls.

### Combining clinical and cellular markers improves accuracy for modelling luma/iva discontinuation

Since the clinical parameters of patients who discontinued due to pulmonary or other adverse effects differed greatly, the model describing associated markers for pulmonary side effects (Table 2) was developed by excluding patients who discontinued due to other adverse effects. Therefore 6 out of 82 (7.3% of total analyzed patients) or out of 17 (35% of patients who discontinued) were excluded from our risk analysis. To distinguish individuals who discontinued treatment due to all adverse effects, a similar model based on clinical factors including all 82 patients was developed. The resulting model included age, neutrophil count, exacerbation rate, CFRD, sinus surgery, *P*. *aeruginosa* and use of oral prednisone and/or nasal budesonide with specificity, sensitivity and accuracy of 95.5%, 50.0% and 83.3%, respectively (Table 6, second column).

**Table 6:** Logistic regression model for distinguishing individuals who discontinued treatment combining clinical and molecular parameters.

Pred/bud	x	x	x	x	x	x	x	x	x	x	x	x	x	x	x	x	x	x
PA M	x	x	x	x	x	x	x	x	x	x	x	x	x	x	x	x	x	x
Sinus Sx	x	x	x	x	x	x	x	x	x	x	x	x	x	x	x	x	x	x
CFRD	x	x	x	x	x	x	x	x	x	x	x	x	x	x	x	x	x	x
Age	x	x	x	x	x	x	x	x	x	x	x	x	x	x	x	x	x	x
PMN	x	x	x	x	x	x	x	x	x	x	x	x	x	x	x	x	x	-
Exac	x	x	x	x	x	x	x	x	x	x	x	x	x	x	x	x	-	-
NK_CXCR2	-	x	-	-	x	-	x	x	-	x	x	x	x	x	x	x	x	x
PMN_CXCR2	-	-	x	-	x	x	-	x	-	x	x	x	x	x	x	x	x	x
M1_CXCR2	-	-	-	x	-	x	x	x	-	x	x	x	x	x	x	x	x	x
NE ELISA	-	-	-	-	-	-	-	-	x	x	-	-	x	-	x	x	x	x
IL-8 ELISA	-	-	-	-	-	-	-	-	x	-	x	-	x	x	-	x	x	x
sTNFR1 ELISA	-	-	-	-	-	-	-	-	x	-	-	x	-	x	x	x	x	x
AIC	33.76	27.76	32.55	34.08	27.3	31.11	28.71	26.38	35.93	26.86	27.14	27.67	28.53	28.55	28.02	30.64	28.58	27.11
likelihood ratio test P value	0.017	0.016	0.012	0.021	0.011	0.006	0.019	0.007	0.022	0.007	0.008	0.009	0.010	0.011	0.009	0.017	0.011	0.008
Specificity	95.50%	95.20%	95.20%	95.20%	95.20%	95.20%	95.20%	100%	95.50%	100%	100%	100%	100%	100%	100%	100%	100%	100%
Sensitivity	50.00%	66.70%	50.00%	50.00%	66.70%	62.50%	50.00%	83.30%	37.50%	83.30%	83.30%	66.70%	100%	100%	100%	100%	100%	100%
Accuracy	83.30%	88.90%	82.80%	82.80%	88.90%	86.20%	85.20%	96.30%	80.00%	96.30%	96.30%	92.60%	**100%**	**100%**	**100%**	**100%**	**100%**	**100%**

The clinical parameters from the most accurate model for identifying individuals who discontinued treatment due to pulmonary and other adverse effects were combined with molecular results to determine which measurements identified treatment discontinuation most accurately (n = 27 patients). The stepwise addition of parameters was performed until accuracy reached 100% (highlighted in bold). Then the least significant clinical parameters were removed one by one until AIC (Akaike information criterion) and P value of the likelihood ratio test reached a minimum without negatively impacting accuracy. “x” and “-”indicate included and excluded parameters, respectively. AIC, Akaike information criterion; CFRD, CF-related diabetes; CXCR2, cell-surface expression of IL-8 receptor beta; ELISA, enzyme-linked immunosorbent assay of proteins in blood plasma samples; Exacerbation, exacerbation requiring intravenous antibiotics; M1, CD14+ CD16- monocytes; NE, neutrophil elastase; NK, NK cell; PA mucoid, mucoid Pseudomonas aeruginosa; PMN, circulating neutrophils; Pred/bud, oral prednisone and/or nasal budesonide.

To further improve the accuracy of distinguishing between the individuals who continued treatment and those who discontinued, we combined levels of CXCR2 and inflammatory blood markers with this clinical data model. These molecular variables were stepwise integrated, and accuracy and goodness of fit of the resulting models compared (Table 6). By combining all CXCR2 values (monocytes, neutrophils and NK cells) with the abovementioned clinical parameters (pred/bud use, sinus surgery, amount of IV antibiotic courses, NTM and mucoid *P*. *aeruginosa* detected in prior year, age and blood neutrophil count at treatment initiation), the accuracy of the model increased to 96.3% (100% specificity and 83.3% sensitivity). Further addition of blood inflammatory markers increased the accuracy to 100%. By removing blood neutrophil count and number of IV antibiotic courses from the model, the goodness of fit was improved without decreasing the accuracy (P = 0.008 by likelihood ratio test). The adjusted odds ratios of the resulting model are shown in Table 7. Due to the smaller size of this cohort, ORs are less significant and might differ compared to values of the aforementioned models with all subjects (n = 82) included.

**Table 7:** A regression model combining clinical and molecular markers accurately distinguishes individuals who discontinued luma/iva treatment in our patient cohort.

Covariates	OR	OR (-95%)	OR (+95%)	P value
Pred/bud use in prior year	13.804	0.446	426.763	0.134
sTNFR1 plasma concentration	2.309	0.286	18.645	0.432
Age at treatment initiation	1.092	0.916	1.301	0.326
CXCR2 on monocytes	1.017	0.998	1.036	0.078
CXCR2 on NK cells	1.006	0.978	1.036	0.670
NE plasma concentration	1.002	0.987	1.018	0.757
CXCR2 on neutrophils	0.991	0.98	1.002	0.092
IL-8 plasma concentration	0.911	0.728	1.139	0.413
Sinus surgery in prior year	0.366	0.001	117.943	0.733
CFRD positive	0.188	0.01	3.467	0.261
Mucoid PA detected in prior year	0.096	0.002	4.934	0.244

P values of clinical parameters, adjusted odds ratios (OR) and lower (-95%) and upper bounds (+95%) of 95% confidence interval are shown. Parameters are sorted from higher to lower OR and dotted line demarcates OR above or below 1. CFRD, CF-related diabetes; CXCR2, cell-surface expression of IL-8 receptor beta; NE, neutrophil elastase; NK, NK cell; Pred/bud, oral prednisone and/or nasal budesonide; PA, Pseudomonas aeruginosa; sTNFR1, soluble tumor necrosis factor receptor 1.

## Discussion

Therapy with the novel CFTR modulator luma/iva has been introduced into the clinic to improve CFTR function in subjects with CF homozygous for the *F508del* CFTR mutation. Along with modest improvement in ppFEV_1_, luma/iva treatment has been shown to slow down disease progression [11] and potentially prolong patient life expectancy [22]. Because a sizeable portion of people with CF who initiated treatment with luma/iva have reported adverse effects leading to discontinued use, we sought to explore markers to distinguish between individuals who tolerated luma/iva and those who discontinued use due to adverse effects. A previous report by Jennings *et al*. [14] analyzing clinical parameters observed no significant effect of ppFEV_1_, CFRD, microbial pathogens or age on luma/iva discontinuation. Interestingly, they reported a higher rate of discontinuation in female patients.

Our retrospective analyses indicate clinical parameters associated with treatment discontinuation and highlight the importance of differentiating its causes. Patients who discontinued due to pulmonary side effects were older and exhibited lower lung function and higher use of systemic and nasal corticosteroids than patients who continued treatment or discontinued due to other causes.

Although the CFF pulmonary care guidelines recommend against the use of inhaled corticosteroids in the absence of asthma and allergic bronchopulmonary aspergillosis [23], steroid use for the treatment of asthma is fairly common in adults with CF [24]. In our program, inhaled budesonide is commonly prescribed for CF-related asthma, while fluticasone propionate is administered as a nasal spray for rhinosinusitis. The clinical indications for prescribing topical or systemic glucocorticoids include severe rhinosinusitis [25, 26], allergic bronchopulmonary aspergillosis [27], severe asthma/bronchospasm that requires treatment beyond typical inhalers, and CF-related arthritis/vasculitis. It is possible that steroid use is a marker for increased inflammation and/or severity of inflammation and comorbidities in these subjects, and that this phenomenon may contribute to the increased frequency of discontinuation due to adverse effects in these subjects. Differences in ppFEV_1_ and ppFEF_25-75_, and the proportion of patients with severe lung disease (ppFEV_1_ ≤ 40%) or a potential indication of small airway disease (ppFEF_25-75_ ≤ 60%) [28] did not reach statistical significance between subgroups. However, their trends further suggest an association of more advanced small airway involvement with the reported chest tightness and increased risk of experiencing other pulmonary adverse effects in response to luma/iva treatment. Interestingly, the use of inhaled bronchodilators was similarly high between the subgroups of patients who continued or discontinued treatment due to various side effects.

Conversely, increased neutrophil count and ppFEV_1_ below baseline seemed to be positively associated with treatment continuation contrary to what one would expect. These factors may be indicative of an exacerbation prior to luma/iva initiation as they were not necessarily measured directly prior to drug initiation. Patients with decreased ppFEV_1_ were evaluated by CF faculty and treated for an acute exacerbation if deemed necessary before luma/iva was initiated. Indeed, 12 out of 20 patients with reported ppFEV_1_ below baseline (11 out of 17 patients with ppFEV_1_ below baseline who continued luma/iva treatment) were deemed to experience an acute exacerbation within 6 weeks prior to treatment initiation and were treated with oral (5/20) or intravenous antibiotics (7/20) before starting on luma/iva. The additional antibiotic treatment and increased airway clearance beforehand could potentially increase the likelihood of tolerating luma/iva treatment. Additionally, 3 out of 20 patients with ppFEV_1_ below baseline slowly uptitrated their dose of luma/iva and of these three, two continued with treatment and one did not tolerate it.

Using only clinical parameters (Table 4) and by excluding patients who discontinued due to other adverse effects, we were able to accurately calculate the risk of discontinuing luma/iva treatment due to respiratory symptoms in 71 of 76 patients in our cohort (93.3% accuracy). We sought to increase the accuracy of modeling treatment discontinuation risk due to all adverse effects by combining clinical and cellular variables. The neutrophil and inflammation specific blood biomarkers IL-8, sTNFR1 and neutrophil elastase were examined based on previous studies investigating changes in their levels during pulmonary exacerbations (reviewed in [29]). In addition, research performed by our group previously identified CXCR2, a receptor for the CF-relevant cytokine IL-8 [18], as a marker of interest in CF individuals undergoing treatment with CFTR modulators [16]. Altered CXCR2 expression on the surface of circulating leukocytes has been observed in a variety of clinical conditions. Decreased expression of CXCR2 is associated with cellular activation and has been reported in septic shock, HIV-1 infection, autoantibody (ANCA)-associated vasculitis, after cardiopulmonary bypass, and others [30–34]. In addition to the current study, increased CXCR2 in leukocytes has been reported in obstructive coronary artery disease and paroxysmal nocturnal haemoglobinuria [35, 36], but the mechanism(s) responsible for this increase have not been identified. CXCR2 antagonists have been tested for efficacy in a number of diseases [reviewed in [37]], including CF [38]. In the study of patients with CF, trends towards a decrease in sputum inflammatory biomarkers were observed with the CXCR2 antagonist treatment; however, there was also an increase in systemic inflammatory markers. Of relevance to the current report, a previously published study examined the influence of CXCR1 and CXCR2 haplotypes on CF lung disease [39]. In this study, certain SNPs correlated with decreased CXCR1/2 expression by neutrophils, CXCR1/2-mediated neutrophil antibacterial effector functions, and lung function. However, the frequency of these SNPs were much lower (<5%) than the frequency of discontinuation reported in the present study; therefore, CXCR1/2 haplotype is unlikely to be the sole mechanism responsible for discontinuation. Future research will explore the role of CXCR2 on leukocytes in CF disease progression and response to CFTR modulation.

Interestingly, markers of systemic inflammation (IL-8, sTNFR1, NE, and leukocyte surface CD63 levels) were not significantly different between patients who discontinued and those who continued the use of luma/iva (Fig 3 and S1 Fig), suggesting that there was no difference in general inflammation present at the time of treatment initiation. Especially given this context, the implications of the increased surface CXCR2 expression on peripheral blood leukocytes from patients who discontinued luma/iva use needs to be further explored. While the levels of IL-8 and lipopolysaccharide are increased in patients with CF (S1 Fig, [40–42]) and both of these molecules have been shown to decrease surface levels of CXCR2 [43, 44], it is intriguing that the surface levels of CXCR2 on leukocytes from CF patients were found to be elevated as compared to levels on cells from non-CF individuals (Fig 3). Research is currently being performed to elucidate the mechanism(s) responsible for the increased levels of surface CXCR2, however, exposure to intermittent hypoxic conditions [45], IL-4/IL-13 [46], and OxLDL [47] *in vitro* has been demonstrated to result in increases in CXCR2 levels and may be relevant to the mechanism(s) involved in the increased CXCR2 observed during CF. Regardless of the mechanism, it is also important to explore the biological relevance of elevated surface CXCR2 levels, which may increase leukocyte migratory capacity and, therefore, their influx into the lung, potentially indicating a more primed neutrophil or state of neutrophil activation [48]. Overall, by combining these molecular markers with the clinical parameters, the discontinuation of 30 out of 30 patients was accurately identified. These results highlight the uniqueness of the distinguishing markers found in this study, and may provide the foundation for future research into outcomes of CFTR dysfunction as well as consequences of CFTR correction in CF subjects.

When interpreting the findings of this study, it is important to note that the number of individuals included in this study was limited. While 150 patients of the Colorado Adult CF Program initiated luma/iva treatment, only 65 had complete records of the prior year, since a large proportion of patients at our center live further away and receive additional care closer to home. This limitation is especially true of the cohort of individuals from which we were able to obtain peripheral blood samples prior to initiation of luma/iva treatment. It is possible that selection bias has been introduced unwittingly, as these patients might be frequenting our program more often and could have more severe disease or require additional treatments for comorbidities compared to patients visiting once a year or less. While the associations described herein may prove useful in identifying subjects more or less likely to tolerate luma/iva, a larger, prospective, multicenter study would be required to validate this model to inform treatment.

Given that the rate of treatment intolerance with lum/iva is higher with clinical initiation than that reported in the phase 3 trials [13–15], there is potential value in the ability to predict intolerable adverse effects of luma/iva in CF subjects. As luma/iva treatment continues to be introduced in a country-by-country fashion, the opportunity will exist to validate the proposed model to predict treatment discontinuation. While this treatment has been clinically available since 2015 in some countries, identifying patients at risk of discontinuation in countries in which the drug has only recently been approved could inform physicians to implement measures to reduce the risk of discontinuation. A recent study has shown that *F508del* patients with advanced lung disease (ppFEV_1_ ≤ 40%) who initiated luma/iva treatment on half-dose with gradual increase to full dose experienced less adverse events and of shorter duration [15].

Additionally, as new CFTR therapeutics are being developed, including tezacaftor/ivacaftor (tez/iva) [49] and next-generation correctors in triple combination [50], these same outcomes and measures can be monitored to examine whether the observed factors associated with discontinuation are relevant to luma/iva alone or CFTR modulators in general. However, recent results from the phase 3 trial for tez/iva indicate less adverse events than reported for luma/iva and less drug-drug interactions, leading to fewer patients discontinuing during the recent trial [15, 49]. Tez/iva showed similar benefits as luma/iva with increase in ppFEV_1_ of 4% and decrease in pulmonary exacerbations by 35% compared to placebo, while luma/iva illustrated a 2.8% increase in ppFEV_1_ and 39% reduction in exacerbation rate [11, 49], respectively. Furthermore, in contrast to luma/iva, pre- and post-dose spirometry performed in a subset of patients in the tez/iva trial demonstrated no post-dose decrease in spirometry [49]. Therefore, the dyspnea and chest tightness observed with luma/iva initiation is likely to be unique to this drug combination or its interaction with concurrent medications. While awaiting the outcome of current trials, our analysis of factors correlating with discontinuation may inform the prescriber to potential adverse effects for the CF subject. By anticipating an increased risk of adverse effects, preventative measures, including slow uptitration of the drug, can be considered.

## Supporting information

S1 Fig Plasma concentrations of inflammatory markers. Plasma concentrations of markers of inflammation, including IL-8 (A), sTNFR1 (B) and neutrophil elastase (C), as measured by ELISA were not significantly different between patients who stopped (stop) and continued (cont.) luma/iva treatment. CF levels were compared to non-CF controls by Kruskal-Wallis test (**P<0.01 line above graph). P values of Dunn’s multiple comparisons test between non-CF controls and each CF subgroup (P values above each dataset) or CF patients combined (P value indicated in the center between both CF groups) indicate significantly lower levels of IL-8 (A) and neutrophil elastase (C) in non-CF controls (*P<0.05, **P<0.01, ***P<0.001). Differences in sTNFR1 (B) levels did not reach statistical significance.
(TIF)

## Abbreviations

AIC: Akaike information criterion
BMI: body mass index
CFRD: CF-related diabetes
CXCR2: cell-surface expression of IL-8 receptor beta
Exac: exacerbation requiring intravenous antibiotics
ppFEV_1_: forced expiratory volume in first second in percent predicted
Iva: ivacaftor/VX-770
Luma: lumacaftor/VX-809
M1: CD14+ CD16- monocytes
MAC: *Mycobacterium avium* complex
MABC: *Mycobacterium abscessus* complex
NE: neutrophil elastase
NK cells: natural killer cells
NTM: non-tuberculous mycobacteria
PA M: mucoid *Pseudomonas aeruginosa*
PA NM: non-mucoid *Pseudomonas aeruginosa*
PMN: circulating polymorphonuclear leukocyte
Pred/bud: oral prednisone and/or inhaled budesonide
Sinus Sx: sinus surgery
sTNFR1: soluble tumor necrosis factor receptor 1
