# Supplementary figures and images for: Corticosteroid use and increased CXCR2 levels on leukocytes are associated with lumacaftor/ivacaftor discontinuation in cystic fibrosis patients homozygous for the F508del CFTR mutation
Source: PLoS One. 2018 Dec 12;13(12):e0209026. doi: 10.1371/journal.pone.0209026 (PMC6291130; doi:10.1371/journal.pone.0209026)

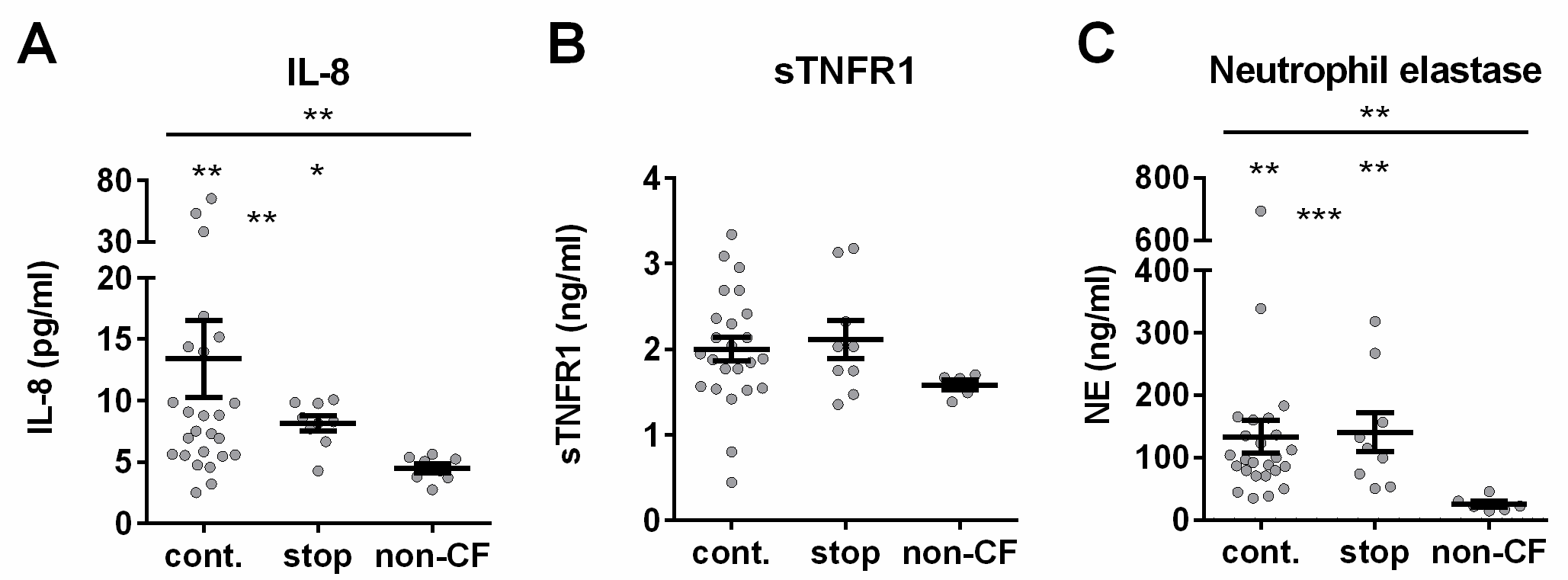

Supplement: S1 Fig — Plasma concentrations of markers of inflammation, including IL-8 (A), sTNFR1 (B) and neutrophil elastase (C), as measured by ELISA were not significantly different between patients who stopped (stop) and continued (cont.) luma/iva treatment. CF levels were compared to non-CF controls by Kruskal-Wallis test (**P<0.01 line above graph). P values of Dunn’s multiple comparisons test between non-CF controls and each CF subgroup (P values above each dataset) or CF patients combined (P value indicated in the center between both CF groups) indicate significantly lower levels of IL-8 (A) and neutrophil elastase (C) in non-CF controls (*P<0.05, **P<0.01, ***P<0.001). Differences in sTNFR1 (B) levels did not reach statistical significance. (TIF) [file pone.0209026.s001.tif]
